# Prevalence of osteoarthritis and clinical outcomes in patients with fractures of the tibial plateau - medium- and long-term analysis

**DOI:** 10.1186/s12891-025-08786-7

**Published:** 2025-05-27

**Authors:** Jon Klasér, Lisa Kotake, Marcus Lindberg, Simon Wigge, Robert Lundqvist, Grzegorz Szczęsny, Przemysław T. Paradowski

**Affiliations:** 1 Department of Diagnostics and Intervention, Division of Orthopedics, Sunderby Research Unit – Norrbotten, Umeå University, Umeå, Sweden; 2 Department of Public Health and Clinical Medicine, Sunderby Research Unit – Norrbotten, Umeå University, Umeå, Sweden; 3 Department of Orthopaedic Surgery and Musculoskeletal Traumatology, Medical University, Warsaw, Poland; 4 Department of Clinical Sciences Lund, Clinical Epidemiology Unit, Orthopedics, Lund University, Lund, Sweden

**Keywords:** Osteoarthritis, Knee, Tibial plateau fracture, Outcome

## Abstract

**Background:**

The incidence of post-traumatic osteoarthritis (OA) following intraarticular knee fractures has been estimated to be relatively high but it varies substantially between different reports. In this study we sought to assess the prevalence of radiographic knee OA secondary to tibial plateau fractures (TPF). The second aim was to report medium- and long-term functional outcomes and investigate whether there were any risk factors associated with these outcomes.

**Methods:**

We retrospectively reviewed documentation of patients who had TPF between 2001 and 2015. The radiographs, clinical characteristics and patient-reported outcome measures (PROMs) scores were evaluated. Presence of radiographic OA was the primary endpoint. The other endpoints were the relationship between OA and different potential predictors as well as the scores in PROMs.

**Results:**

The study involved a total of 130 patients including 114 who were radiographically examined at mean follow-up time of 10 years (range 4.6–19.3 years). Radiographic OA was present in 50% of patients (34% in the injured knee and 16% in both knees). Having OA in the contralateral knee increased the odds to develop OA in the index knee (OR = 4.8; 95%CI 1.6–4.1 in the crude model and OR = 6.6; 95%CI 1.8–23.5 in the model adjusted for age, sex, BMI, fracture type and treatment method). The occurrence of OA was associated stronger with medial or bicondylar TPF than with lateral condyle TPF (OR = 2.8; 95%CI 1.2–6.1 in the crude model and OR = 3.4; 95%CI 1.4–8.6 in the adjusted model). The KOOS scores were significantly lower in patients with OA than in those without OA in the index knee in all the KOOS subscales (*p* < 0.007), except for the KOOS Symptoms (*p* = 0.362). The EQ-5D-5L index score was significantly higher in patients without OA in the index knee compared to those with OA (*p* = 0.015).

**Conclusion:**

Radiographic OA following TPF occurred in 50% of knee joints. The odds for knee OA were highest after medial or bicondylar fractures. Patients with OA in the index knee had lower scores in both condition-specific and generic PROMs than subjects without OA, which indicates that TPF may contribute to the development of both OA disease and illness.

**Trial registration:**

The trial *was registered retrospectively* on June 4, 2024 *on ClinicalTrials*.gov (registration number: NCT06451510).

## Background

Osteoarthritis (OA) is one of the most common causes of pain and disability in the elderly [1]. The disease can affect any joint, but is most common in knees, hips and hands [2]. The incidence of OA increases with age [3]. The prevalence is currently estimated to be nine percent in the adult population [4]. Symptomatic knee OA occurs in approximately 10 percent of individuals over the age of 55 [5]. Every year around three percent of middle-aged people receive an OA diagnosis [6].

There is an array of internal (such as female sex and genetic predisposition) and external, predominantly joint-specific (such as injury or joint overload) factors that increase the risk of both OA disease (defined as structural changes demonstrated in imaging studies) and illness (patient-relevant symptoms of OA) [7–9].

Post-traumatic OA accounts for 12 percent of all OA [10]. Post-traumatic OA is a consequence of an initial impact to the cartilage and/or chronic loads resulting from joint incongruity, instability and malalignment that subsequently activates a cascade of inflammatory and metabolic changes [11–13].

Most research done on post-traumatic OA in the knee joints focuses on the consequences to injuries of ligament and menisci [14–18]. In contrast, the number of studies focused on post-traumatic OA resulting from fractures is small. Intraarticular tibial plateau fractures (TPF) make up one percent of the total number of fractures and their incidence is 10.3 per 100,000 people annually [19]. The fracture is an effect of a combination of an axial force, e.g. fall from a height, and a varus or valgus directed force against the knee joint e.g. blow from side. This causes angulation of the knee joint making the femoral condyle press heavily on the tibial plateau. The injury can occur in both older patients with osteoporosis as well as younger ones exposed to high-energy trauma, often associated with ligament and/or meniscus injuries [20]. Displaced TPF are treated operatively in order to restore the joint anatomy and to create conditions for healing and early mobilization. OA following TPF develops from both incongruence and residual axial deformation of the lower extremity [21, 22].

The fact that frequency of OA increases with time after TPF was reported already in 1955 [23]. Another historical study reported that the incidence of radiographic OA was 56 percent at two to five years and as much as 78 percent at 20-years follow-up [21]. More recent investigations reported slightly lower incidence of post-traumatic OA ranging from 21 to 44 percent [24–28].

Nevertheless, most of these studies had relatively short follow-up time and were carried out on small study samples, one contained as little as 33 [26] and another 46 patients [27]. The studies described the results of surgical treatment performed mostly with methods which are no longer in use. In a recent study, Snoeker et al. have estimated that the risk of OA development in patients with intraarticular knee fractures is six times greater than in patients without an earlier knee injury [29]. OA can probably be prevented by restoring the joint mechanical axis and joint congruence which in turn can help regain joint stability [30, 31].

The natural course of OA following TPF has not yet been properly described. Since the rate post-traumatic OA progression varies between individual patients and neither clinical symptoms nor functional outcomes correspond to radiographic findings, long-term evaluation is necessary for the assessment of post-traumatic OA progression, especially at an individual level [32, 33].

To the best of our knowledge, no study regarding the associations between such factors as fracture type, treatment method and OA rate as a consequence of TPF has been published. We expect that this expertise can add more information for a better understanding how to minimize the risk of post-traumatic OA, and can contribute to identifying factors that may influence patient outcome over time.

The aim of the study was thus to *i*) assess the prevalence of radiographic knee OA secondary to TPF, and *ii*) investigate whether the development of OA following TPF is related to potential predictive factors such as gender, age, body mass index (BMI) and fracture pattern. Furthermore, this study strove to *iii)* investigate the functional outcome after TPF and to gain an increased understanding as to how TPF affects patients in the medium- and long-term, both after surgical- and non-surgical treatment.

## Methods

### Study design

This project was a part of the observational study *Knee Osteoarthritis in the Region of Norrbotten* (KORN) registered retrospectively on *ClinicalTrials*.gov (registration number: NCT06451510) on June 4, 2024.

### Study cohort

The study population consisted of patients from the Swedish region of Norrbotten (northern part of Sweden) who had undergone TPF between the years of 2001 and 2015. All subjects were retrospectively identified by a computer-assisted search of the local medical records database *Vård Administrativt System* (VAS), using the ICD-10 diagnostic codes for proximal tibia fracture (open and closed) (S82.1, S82.8 and S 82.9). All records obtained were then screened manually by four authors (JK, LK, ML and SW) and inappropriate and/or improperly assigned cases were excluded.

**Fig. 1:**
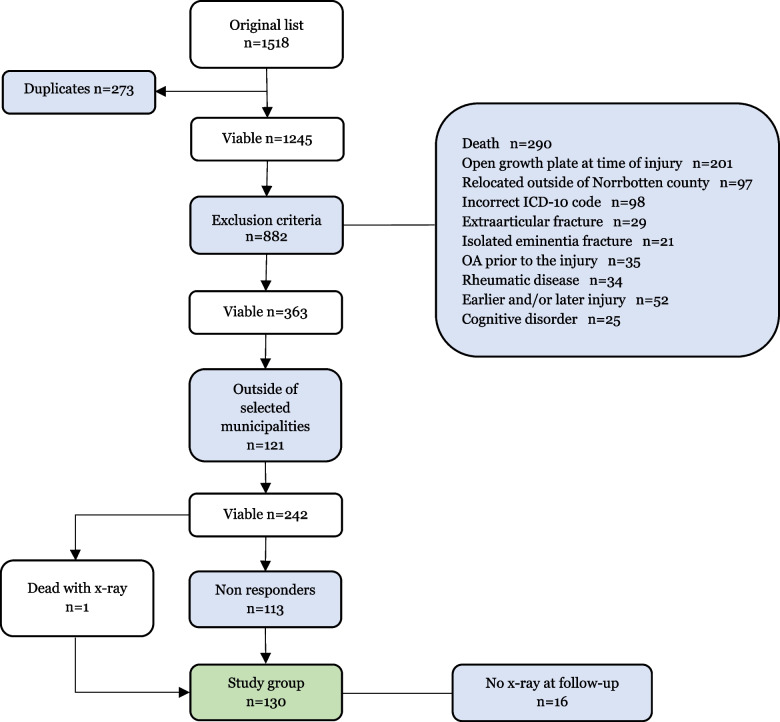
Flowchart presenting the study group formation

The initial sample contained 1518 patients. Each patient’s medical history was reviewed and the following exclusion criteria were applied: 1) death without bilateral weighted X-ray minimum 5 years post injury, 2) open growth plate in distal femur and/or proximal tibia, 3) extraarticular or isolated eminentia fracture, 4) OA prior to injury, 5) having a rheumatic disorder involving joints, 6) earlier and/or later severe injury to the index knee or 7) having cognitive disorders disqualifying from the study (Fig. 1).

Due to a lower accessibility of patients from the distant parts of Region Norrbotten, the study was limited to those subjects who lived in the municipalities of Luleå, Boden, Piteå, Älvsbyn and Kalix (located on the coastline of the Gulf of Bothnia), and thus were available for both clinical and radiographic assessment.

This generated a pool of 242 potential subjects who were individually contacted on phone numbers provided by the VAS. A total of 130 subjects were recruited for the study, even though 20 of them did not participate in the radiographic examination at follow-up (Fig. 1).

### Data collection

All patients were evaluated radiographically after the fracture and for those who were operated on, additional radiography was acquired postoperatively. Radiographs that were taken at different time points after injury in order to follow-up the bone healing were not analyzed.

During the follow-up appointment (between September 2020 and April 2021), the patients were required to undergo clinical and radiological examinations. In addition, all subjects completed a demographic survey and reported on their knee-specific as well as general health status.

Self-reported weight and height was obtained at follow-up from 130 patients who formed the study group.

### Radiographic imaging

Pre- and postoperative radiographic assessment consisted of non weight-bearing posteroanterior and lateral images of the knee joint. Radiological characteristics of all TPF was evaluated according to the Schatzker classification [34].

The radiographic follow-up assessment consisted of weight-bearing posteroanterior and lateral views of both tibiofemoral joints. The images were obtained with the knee in 15 degrees of flexion. The body weight was equally distributed between the two legs and the big toes of both feet and the front of the thighs were placed in contact with the front plate of the plexiglass positioning frame. The external rotation of the feet was fixed at 10 degrees using a V-shaped foot angulation frame support. For optimal plateau alignment the central radiographic beam was guided by fluoroscopy with the beam angled at ~ 10 degrees caudally. The examination was carried out with the Siemens Basic Radiological System (model Ysio or Rax, Siemens Healthcare GmbH, Erlangen, Germany). Sectra Workstation UniView, version 21.2 (Sectra AB, Sweden, 2019) was used in the evaluation of all x-rays.

The date of radiographic examination was considered the follow-up date.

Four independent investigators (JK, LK, ML and SW), blinded to the clinical data, graded all radiographs. To ensure consistent assessment and avoid a drift throughout the reading session, specific radiographs within the study material were selected as the reference material and read repeatedly. If classification differed between the readers, the radiographs were re-read and discussed until a consensus was achieved. If the readers were still in disagreement, the senior investigator (PTP) was consulted.

All radiographs were assessed for joint space narrowing (JSN) and osteophytes using the 4-point scale according to the Osteoarthritis Research Society International (OARSI) atlas [35, 36] (ranging from 0 to 3, where 0 stands for no presence of osteophytes or no JSN). Additional analysis of radiographs was performed using the Kellgren-Lawrence (KL) classification [37].

### Outcome measures

Condition-specific scores were assessed using the Knee injury and Osteoarthritis Outcome Score (KOOS). KOOS is a self-administered knee-specific questionnaire consisting of 42 items in five subscales including: Other symptoms (7), Pain (9), Activities of daily living (ADL) (17), Sports and recreation (Sport/Rec) (5) and Knee related quality of life (QoL) (4) [38]. A normalized score from 0 (extreme problems) to 100 (no problems at all) are calculated separately for each of the five subscales.

General health related quality of life was assessed by the EuroQol 5-Dimension 5-level (EQ-5D-5L) tool [39]. The EQ-5D has been shown to have acceptable reliability and validity for multiple musculoskeletal conditions [40] and limb injuries [41].

The EQ-5D-5L questionnaire consists of five questions exploring five dimensions: mobility, self-care, usual activities, pain/discomfort, and anxiety/depression. Each dimension has five response levels, ranging from “no problems” to”extreme problems”.

The answers are assembled to a five-digit health state reflecting the score in each dimension (in total 3125 states). An EQ-5D-5L index of 1.0 indicates the best possible health state and 0.0 denotes death. In our study, the EQ-5D-5L index value was calculated with answers from each domain according to the Swedish experience-based Time Trade-Off (TTO) method [42]. The Swedish value set of the EQ-5D-5L ranges from 0.2433 (the worst possible health state 55,555) to 0.9755 (best possible health state 11,111).

The self-reported questionnaires were generally filled in immediately after clinical evaluation and submitted before leaving the clinic, although some were completed at home and returned by mail.

### Primary endpoint

The presence of radiographic OA at follow-up was considered the primary endpoint of this study. We used previously proposed criteria to make a diagnosis of radiographic OA in the tibiofemoral joint using the OARSI atlas. These criteria were: *i*) JSN grade in any of the two tibiofemoral compartments ≥ 2, *ii*) osteophyte score in any of the two tibiofemoral compartments ≥ 2 or *iii*) JSN grade 1 in combination with a grade 1 osteophyte in the same tibiofemoral compartment. This cut-off approximates tibiofemoral OA defined as grade 2 changes with either “definite osteophytes and possible narrowing of joint space” or only “definite osteophytes” [43] based on the commonly used KL scale [44].

All subjects who did not have the diagnosis of radiographic OA at time of injury were pre-supposed to not have changes regarding JSN prior to the study.

End stage knee OA was classified as presence of a grade 3 in JSN or if the patient had undergone joint replacement.

### Secondary endpoints

The secondary endpoint of the study was the relationship between the development of radiographic OA in patients who had undergone TPF and such potential factors as gender, age, body mass index (BMI) and fracture pattern. Additional secondary endpoints were the scores in all five subscales of the KOOS and the scores in the EQ-5D-5L at medium- to long-term follow-up after TPF.

The reference KOOS scores in respective subscales were defined as the median value of the age- and gender-matched subjects from the Swedish population [45]. The reference score for the EQ-5D-5L index was defined according to the age- and sex-matched population reference data [46].

### Statistical analysis

Descriptive statistics were used to describe sociodemographic and clinical characteristics at follow-up. Continuous outcomes were given as mean [standard deviation, SD] values. Data were checked for normality of distribution using the Kolmogorov–Smirnov test and tests for skewness and kurtosis. Since the data were not normally distributed, the Mann–Whitney U test was used for the assessment of comparisons between the groups. Binary data in 2 × 2 tables were evaluated by Fisher’s exact test. A two-tailed *p*-value was considered statistically significant if less than 0.05.

In order to identify factors that potentially contribute to the development of KOA following the TPF, logistic regression models were created. A number of independent continuous and categorical variables were examined. Logistic regression analysis was performed in 114 subjects who had radiographic examination. No missing values in variables assessed were found.

Subjects were divided according to sex and BMI. Individuals with a BMI under 25 were categorized as normal, those with BMI between 25 and 29.9 were considered overweight, while those with a BMI of 30 or more were considered obese [47]. With the intention to avoid subjectively chosen limits and to be able to deal with possible non-linear effects, we categorized individuals into tertiles based on their age (T1: < 53.9 years, T2: 54–66.9 years, and T3: > 67 years). In addition, the patients were divided into two subgroups according to the fracture type (lateral condyle fractures belonging to the Schatzker type I, II and III and medial or bicondylar fractures belonging to the type IV, V and VI), and evidence of OA in the contralateral knee.

Type of treatment was dichotomously classed as operative (71 subjects, 55%) and non-operative (59 subjects, 45%).

Each independent variable was analyzed in an unadjusted and an adjusted model. The adjustment was made for age, sex, BMI, fracture type, and presence of OA in the contralateral knee. The odds ratio (OR) estimates with 95% confidence intervals (95% CIs), and results from the likelihood ratio test, expressed as p values, were based on the models. Model fit was examined using the Hosmer–Lemeshow test [48].

Analyses were performed with the IBM SPSS Statistics software package, version 27 (IBM Corp., Armonk, NY, USA).

### Ethics approval and consent to participate

The study was conducted in accordance with the ethical standards of the institutional and/or national research committee and with the 1964 Helsinki declaration and its later amendments or comparable ethical standards. The study was approved the Research Ethics Committee of Umeå University and acquired ethical approval (DNR: 2016/20–31) with regards to access to patient medical records, clinical examinations and radiographic procedures of included patients as well as their later assessment. In addition, the patient exposure to radiation was tested and approved by the Radiation Protection Committee for Region Norrbotten (approval dated October 13, 2020).

The patients were informed in writing and orally by the study personnel, and a written informed consent was obtained from all subjects. Participation was voluntary, and withdrawal was possible at any time. All patients signed and personally dated the informed consent forms at admission to hospital, before participating in the study.

## Results

### Subject characteristics

The study group consisted of 130 subjects (55 men and 75 women) with the mean age at follow-up of 59 years (median 59.8, range 22.9–95.9). Women were significantly older than men (median age 62.2, range 22.9–90.4 vs. 53.6 years, range 23.0–95.9, *p* = 0.006). The mean follow-up time was 10.0 years (median 9.7, range 4.6–19.3) (Table 1).

**Table 1:** Characteristics of the study group

Characteristic	All subjects *N* = 130	Men *N* = 55	Women *N* = 75
Mean age [SD], years			
At injury	49.0 [15.2]	45.0 [15.5]	51.9 [14.5]
At follow-up	59.0 [15.2]	55.2 [15.4]	61.8 [14.6]
Follow-up time	10.0 [3.6]	10.2 [3.5]	9.9 [3.7]
Fracture type N (%)			
I	29 (22)	9 (7)	20 (15)
II	28 (22)	16 (12)	12 (9)
III	25 (19)	7 (5)	18 (14)
IV	18 (14)	10 (8)	338 (6)
V	17 (13)	5 (4)	12 (9)
VI	13 (10)	8 (6)	5 (4)
Side injured, N (%)			
Right	61 (47)	28	33
Left	68 (52)	27	41
Bilateral	1 (1)		1
Treatment, N (%)			
Conservative	59 (45)	22	37
Operative	71 (55)	33	38
BMI, mean [SD]	27.9 [5.8]	28.5 [6.2]	27.4 [5.4]

*Abbreviations*: *BMI* body mass index, *SD* standard deviation

Seventy-one patients (55%, 33 men and 38 women) were operated on, whereas conservative treatment was applied in 59 patients (45%, 22 men and 37 women).

In the group as a whole, the mean BMI at the follow up assessment was 27.9 (median 26.7, range 18.0–54.9) with no significant difference between men and women (*p* = 0.469). The study group consisted of 91 patients in normal BMI range (70%), 23 patients with overweight (18%) and 16 patients with obesity (12%).

Eighty-two out of 130 patients (63%) had TPF of type I, II or III according to Schatzker classification. The most complex fracture patterns, type V and VI, were observed in 17 (13%) and 13 subjects (10%), respectively. There were no significant differences in Schatzker type distribution between men and women. Most patients with fractures belonging to types I-III (49/82 subjects, 60%) were treated conservatively. Almost all patients with the most complex fracture pattern were operated on. The only patient with Schatzker type V who was treated conservatively had a totally nondisplaced fracture.

### Primary endpoint

Radiographic examination at follow-up was performed in 114 patients (85% of the study group, 48 men and 66 women) with the mean age at follow-up of 59 years (median 60.3, range 23.0–86.7) (Table 2).

**Table 2:** Presence of OA after TPF at follow up in the index and in the contralateral knee

Characteristic	All subjects	Men	Women
Knee X-ray at follow-up, N (%)	114 (100)	48 (42)	66 (58)
Mean age at follow-up [SD], years	59.1 [14.2]	53.5 [14.6]	63.2 [12.5]
OA at follow-up			
Index knee	57 (50)	22	35
Contralateral knee	23 (20)	4	19
Bilateral	18 (14)	4	14

*Abbreviations*: *OA* osteoarthritis, *TPF* tibial plateau fracture, *SD* standard deviation

Fifty-seven patients (50% of those who had x-ray at follow-up) developed OA in the index knee of which 39 (34%) had OA only in the index knee and 18 (16%) in both knees. Five patients (4%) developed OA only in the contralateral knee joint. End-stage knee OA was present in five patients (4%).

There were no statistically significant differences between men and women regarding the number of patients who developed OA.

### Secondary endpoints

#### Potential predictors of radiographic OA

Multivariable logistic regression analysis showed that having OA in the contralateral knee joint markedly increased the odds for developing OA in the index knee. This association was observed in both the crude (unadjusted) model (OR = 4.8; 95%CI 1.6–14.1) and in the model adjusted for age, sex, BMI and fracture type (OR = 6.6; 95%CI 1.8–23.5) (Table 3).

**Table 3:** Odds ratios for the development of OA in the index knee after TPF by patient characteristics and selected predictors in an unadjusted (crude) model and the model adjusted for age, sex, BMI, presence of OA in contralateral knee and fracture type

Factor	Number of cases		Unadjusted		Adjusted	
	N	%	OR	95% CI	OR	95% CI
**Gender**						
Man^a^	48	42				
Woman	66	58	1.3	0.6–2.8	0.9	0.4–2.4
**Age (years)**						
< 53.9^a^	37	32				
54–66.9	40	35	1.1	0.4–2.6	0.7	0.2–2.0
> 67	37	32	2.2	0.9–5.5	1.5	0.5–4.8
**BMI (kg/m**^**2**^**)**						
< 25^a^	81	71				
25–30	21	18	2.5	0.9–6.8	3.5	1.1–11.0
> 30	12	11	1.8	0.5–6.0	1.2	0.3–5.2
**OA in contralateral knee**						
No^a^	91	80				
Yes	23	20	4.8	1.6–14.1	6.6	1.8–23.5
**Fracture type**^**b**^						
I—III^a^	73	64				
IV—VI	41	36	2.8	1.2–6.1	3.4	1.4–8.6

*Abbreviations*: *OA* osteoarthritis, *TPF* tibial plateau fracture, *BMI* body mass index, *OR* odds ratio, *95% CI* 95% confidence interval

^a^Reference category; ^b^Fracture type according to Schatzker classification

It was also examined whether there is an association between the presence of OA in the index knee and the fracture pattern. OA has the strongest relationship with medial or bicondylar TPF (type IV-VI, OR = 2.8; 95%CI 1.2–6.1 in the crude model and OR = 3.4; 95%CI 1.4–8.6 in the adjusted model) (Table 3).

No significant association between the presence of radiographic OA and sex, age or BMI was observed.

All 130 patients who formed the study group completed both the KOOS and EQ-5D-5L questionnaires.

#### KOOS

The mean KOOS scores at follow-up were highest for the subscales Pain and ADL (84 and 86.7 respectively) and lowest for the subscale Sports/Rec (56.6) (Table 4).

**Table 4:** KOOS scores and EQ-5D-5L index scores and the number of subjects who scored above the age- and sex-matched KOOS reference values and above the cut-off for the EQ-5D-5L index

Characteristic	All subjects	Men	Women
KOOS scores at follow-up, mean [SD]			
Pain	84.0[16.6]	85.5 [15.8]	82.9 [17.3]
Symptoms	62.7 [11.4]	61.6 [11.5]	63.4 [11.4]
ADL	86.7 [17.3]	88.2 [15.5]	85.7 [18.4]
Sport/Rec	56.6 [33.5]	59.4 [34.1]	54.5 [33.1]
QOL	64.9 [24.6]	64.8 [25.3]	64.9 [24.2]
KOOS scores ≥ reference values, N (%)			
Pain	60 (46)	21	39
Symptoms	2 (1.5)	1	1
ADL	61 (47)	21	40
Sport/Rec	41 (32)	20	21
QOL	41 (32)	17	24
EQ-5D-5L index, mean [SD]	0.882 [0.112]	0.877 [0.12]	0.884 [0.107]
EQ-5D-5L index, ≥ reference values N (%)	72 (55)	28	44

*Abbreviations*: *KOOS* Knee injury and Osteoarthritis Outcome Score, *EQ-5D-5L* EuroQol 5-Dimension 5-level, *ADL* activities of daily living, *QOL* quality of life, *SD* standard deviation

Men scored significantly higher than women in the KOOS subscale ADL (88.2 vs 85.7, *p* = 0.05). No remarkable mean score difference between men and women was observed in other subscales.

The number of patients who exceeded the age- and sex-matched population reference score in different KOOS subscales was highest in the subscale KOOS Pain (60 subjects, 46%) and KOOS ADL (61 subjects, 47%). There were one male patient and one female patient who exceeded the reference score in the KOOS subscale Symptoms. Predominance of women who exceeded the reference scores for the KOOS subscales Pain (39/75 vs 21/55 for men) and ADL (40/75 vs 21/55) was not statistically significant (*p* = 0.15 and *p* = 0.11, respectively).

There were 24 men and 28 women who did not exceed the reference score in any of the KOOS subscales.

**Fig. 2:**
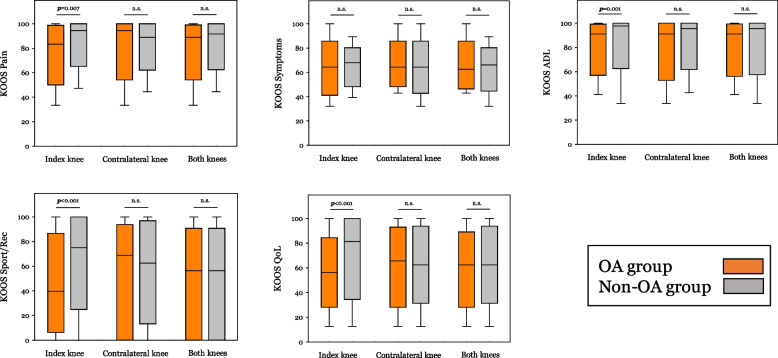
Comparison of the KOOS scores in patients following tibia plateau fracture. Each diagram shows the index values in patients with (orange boxplots) and without osteoarthritis (OA) (gray boxplots) in the index knee, contralateral knee and in both knees, in all five KOOS subscales. The whiskers represent minimum and maximum values while the horizontal line inside the box is the median value. The 25 th and the 75 th percentiles mark the upper and lower end of the box. *P* value is given over the boxes

The KOOS scores were found to be significantly lower in patients who developed OA than in those without OA in the index knee in all the KOOS subscales except for the KOOS Symptoms (*p* = 0.362) (Fig. 2). The differences in the KOOS scores in patients with OA and in those without OA in the contralateral knee and in both knees were not statistically significant.

No statistical differences were observed between the KOOS scores in all the subscales in patients with OA only in the index knee and patients who had OA in both knees or only in the contralateral knee (data not shown).

#### EQ-5D-5L

Values of the EQ-5D-5L index ranged at follow-up from 0.452 (health state 33434) to 0.976. The best possible health state (health state representing no problems at any dimension, 11111, 0.976 according to the Swedish TTO value set) was reported by 29 patients (11 men and 28 women).

The mean health status index at follow-up was 0.882 [SD 0.112]. Men reported a mean of 0.877 [0.12] and women 0.884 [0.107]. Seventy two out of 130 patients (55%, 28 men and 44 women) had an index value that exceeded the age- and sex-matched population reference values. Among those who had the index score over the population reference value 35% (26 out of 57 subjects) had and 65% (37/57) had no post-traumatic OA. This difference was not statistically significant (*p* = 0.059, data not shown).

The EQ-5D-5L index score was significantly higher in patients without OA in the index knee compared to those with OA (*p* = 0.015).

**Fig. 3:**
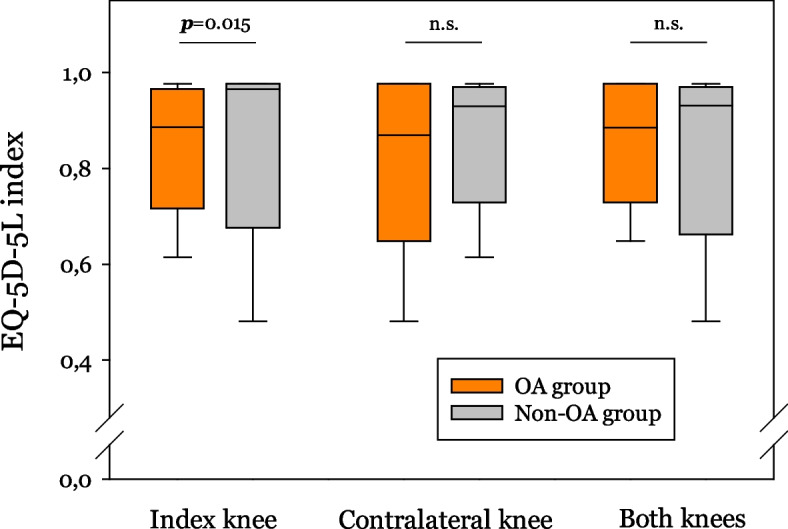
Comparison of the EQ-5D-5L index in patients following tibia plateau fracture. Each diagram shows the index values in patients with (orange boxplots) and without osteoarthritis (OA) (gray boxplots) in the index knee, contralateral knee and in both knees. The whiskers represent minimum and maximum values while the horizontal line inside the box is the median value. The 25 th and the 75 th percentiles mark the upper and lower end of the box. *P* value is given over the boxes

No statistical difference between the EQ-5D-5L index scores was observed in patients with and without OA in the contralateral knee or in both knees (Fig. 3). The index values were not different in patients with OA only in the index knee and those who had OA in both knees or only in the contralateral knee joint (data not shown).

## Discussion

In this study we presented the results of a long-term radiographical and clinical outcome assessment of patients with earlier TPF. As expected, we confirmed that radiographic OA was substantially more frequent in the previously injured (index) knee joint than in the contralateral knee. We found that post-traumatic OA was present in the index knee joint in one third of all patients examined. Additional 16 percent of subjects acquired features of radiographic OA in both, the index and the contralateral knee, and five patients (4%) only in the contralateral knee. This finding shows that the disease in one fifth of the patients examined was developed independently of injury and might suggest a general predisposition for OA disease.

For assessment of radiographic OA we used the classification of the Osteoarthritis Research Society International (OARSI) in the form of a radiographic atlas published in 1995 [35] and then revised in 2007 [36]. We have however made additional assessment using the most common Kellgren-Lawrence (KL) radiographic OA scale [37]. Both, the KL scale and the simplified form of the OARSI classification based on the JSN assessment were found to be valid and suitable for clinical practice [49, 50]. However, since the OARSI atlas classification puts more emphasis on JSN and describes the changes in separate compartments, it has been assumed that it is more sensitive for diagnosing OA and assessing the severity of the disease [51]. That has not been confirmed in our study. According to the KL classification with OA defined as grade 2 changes (corresponding to either “definite osteophytes and a possible narrowing of the joint space” or only “definite osteophytes”), 66 patients (58%) had evidence of radiographic tibiofemoral OA in the index knee of which 30 (26%) had OA in both knees. Eight patients (7%) developed OA only in the contralateral knee joint. A significant prevalence of knees with OA when assessed with the KL method (104 vs 80 knees with OA) may be caused by difficulties with understanding of grade 2 changes in the KL classification, especially concerning the joint space narrowing [52]. However, our study was not aimed at comparing the two methods of OA diagnostics and these results must be interpreted with caution.

The prevalence of post-traumatic OA following TPF in our series corroborated previous findings. Post-traumatic OA is currently estimated to occur in approximately 23% to 36% of subjects [53–56], of which a majority have only mild to moderate changes [53]. Small discrepancies in the study results are likely due to differences in follow-up time and/or different criteria for the diagnosis of OA. Contrary to expectations, the prevalence of OA in the uninjured knee (16% of patients with OA in both knees and additional 4% only in the contralateral knee, which makes 20%) in patients assessed in our study was higher than OA prevalence in general population. It has earlier been reported that the prevalence of knee OA in Europe was estimated to be between 13 and 15% [57, 58]. The discrepancy between these studies and our results may be caused by the method of OA assessment or differences in the characteristics of the study cohort. Still, it has been observed that injury can accelerate the progression of OA not only the index, but even in the contralateral knee. The alterations in joint loading on the injured side can lead to biomechanical compensation in the contralateral knee, which eventually leads to overuse and degradation. Nevertheless, it is suspected that the mechanisms that result in OA progression of the contralateral limb are different than primary knee OA progression [59].

It has previously been assumed that the incidence of OA is predicted by the severity of fracture and joint malalignment [30, 60]. In our sample more severe medial or bicondylar fractures belonging to the Schatzker type IV, V and VI type were diagnosed in 37% of patients, whereas 63% of subjects had lateral unicondylar TPF. It would certainly be interesting to assess the odds of OA development in relation to each of the fracture type. However, too many variables in logistic regression models in a relatively small patient sample would constitute a risk of overfitting and bias. Patients were thus assembled into two larger subsets, one comprising lateral unicondylar fractures, the other medial or bicondylar TPF. As expected and in line with earlier reports [28, 56, 61] the odds of OA development in our study were three times larger in subjects who had undergone either medial or bicondylar TPF (Schatzker type IV, V and VI) than in those with lateral unicondylar fractures.

We found that having OA in the contralateral knee was associated with nearly fivefold (or even nearly sevenfold in the regression model adjusted for age, sex, BMI and fracture type) increase of odds of developing OA in the index knee. This observation may indicate that post-traumatic OA disease depends partly on factors not associated with the actual injury, but probably factors of environmental or genetic nature. Though all three being accepted as great risk factors for the general population, older age, female sex and high BMI [62, 63] did not show any statistical significance in our series, presumably due to the limited sample size.

In this study we also assessed long-term subjective outcomes following TPF with the use of PROMs including both the condition-specific measure KOOS and generic measure EQ-5D-5L. As expected, the patients with OA in the previously injured knee reported more pain and greater difficulties in performing both daily and sport-related activities than those without OA which was expressed as lower scores in the KOOS subscales Pain, ADL and Sport/Rec. There was, however no difference in mean scores in the KOOS subscale Symptoms between the subjects who did or did not have OA in the index knee. Symptoms such as joint stiffness and swelling are probably not very sensitive to change over time since the weakest responsiveness of the KOOS Symptoms subscale had previously been observed in several validation studies [64, 65].

As the TPF could have long-term consequences beyond physical conditions, including psychological and social conditions, we also assessed the health-related quality of life. This could be difficult to capture using one disease-specific instrument. Therefore in order to gain a more comprehensive approach, an assessment of both generic and condition-specific quality of life is recommended [66].

To the best of our knowledge, our research is the first to assess the medium- and long-term patient-reported outcomes following TPF. Previous studies on the outcomes after injuries to the knee joint addressed almost exclusively ligament- and meniscus tears and therefore included mainly younger subjects. It was reported that sport-related knee injury had an impact mostly on condition-specific quality of life [66, 67]. The relatively poorer outcomes for generic quality of life measures were observed in subjects who had radiographic or symptomatic features of OA [68, 69]. In addition, individuals with OA (without specified history of injury) reported worse generic and condition-specific quality of life mean scores compared to healthy individuals [66].

Our findings showed that patients with post-traumatic OA in the index knee had worse scores in both generic measure EQ-5D-5L and in the condition-specific KOOS QoL subscale compared to those without OA. The previously formulated suggestion that risk factors for OA illness may differ from those for OA disease [66], although commonly accepted, cannot be therefore confirmed by our results.

Since we had no control group in this study, we could compare the results obtained only with the age- and sex-matched population reference values, which however gives even more comprehensive and useful data. We observed that 55% of patients scored above the reference values in the generic EQ-5D-5L. That figure was significantly higher than the percentage revealed by the condition-specific KOOS QoL subscale (32%, *p* < 0.001). Since only half of the study group had signs of post-traumatic OA, it is natural that our assessment with condition-specific measures appeared to be more discriminating.

No significant difference in any of the KOOS subscales or in the EQ-5D-5L index was reported between patients with and without OA in the contralateral knee or in both knees. A lack of statistical significance may be caused by the lower number of patients with OA outside the injured knee and a lesser severity of disease in patients who had OA.

The present study is one of few that assess X-ray images and captures the perspective of patients who have undergone a TPF with regard to development of post-traumatic OA. The study was carried out with a relatively long follow-up time during which, despite subtle differences in operations technique and some modifications and advancements in plate osteosynthesis, treatment principles remained effectively the same. The study sample reflects the demographic distribution of TPF in the Danish population [70].

We acknowledge several limitations of the study. We retrospectively reviewed data from a single database where the identification of patients was based on procedural coding. Some cases may then have been missed due to inaccuracy of diagnoses, in particular in the beginning of the 2000’s when we used the earlier coding system KSH97. The precision and completeness of diagnoses increased when the new ICD-10 coding was introduced in Sweden in 2011. Some of the patients studied were however, injured when the ICD-10 coding was still new and the sample could be still affected by a potential selection bias if fracture diagnoses were incorrectly attributed to the new, expanded codes. A second limitation is that we could not assess potential confounders that varied over time. We did not have information on the height and weight at injury, so BMI was measured only at follow-up.

Another limitation of our study is the small size of the study sample. The patient count was lower than expected due to the Covid-19 pandemics. Due to the logistic difficulties and low accessibility we excluded patients with TPF who lived in remote municipalities of the Region of Norrbotten. The population pattern in the municipalities that were not studied is however similar concerning both the urbanization as well as age and gender distribution. Since about 80% of TPF happened in the coastal area of included municipalities, we believe that our selection did not affect the final results. The response rate of 54% is relatively high for long-term follow-up of an orthopedic trauma population. Since the additional analysis concerning sex, age and fracture type and time from injury did not reveal any significant differences between non-respondents and respondents, we do not expect a significant non-response bias in our study group. The number of subjects studied appeared to be sufficient to detect factors potentially predisposing to the development of both OA disease and OA illness in the group as a whole. However, the study group was not large enough to either exclude the youngest patients or to extract smaller age groups in order to assess the differences between them. In particular, it would be interesting to investigate whether high-energy injuries are more frequent in younger subjects and whether such injuries result in more complex fracture patterns and, consequently, in higher prevalence of OA. A larger database of a variety of participating hospitals or, even better, a fracture register database would give more representative results.

Although all participants were examined within a relatively short time frame, the follow-up time varied substantially between individual cases. We assumed that five-year follow-up was long enough for patients to develop OA. However, since we did not assess the radiographic progression, the categorized diagnosis of OA did not capture all tendencies in patients who were close to fulfilling the criteria for radiographic OA and who possibly could meet them with a longer observation time.

For obvious reasons, radiographs taken immediately after the injury were neither weight-bearing nor bilateral, which made it impossible to assess JSN the same way we assessed patients at follow-up. We could possibly miss some cases with JSN corresponding to grade 2 change according to the OARSI atlas which is sufficient to diagnose OA. In addition, all of the main data collectors were physicians in training, likely to make errors in grading or physical assessment. Furthermore, the presence of deformities after operation and/or injury gave some difficulties in scoring. Radiographs were verified by an experienced reader to minimize possible mistakes, but they could not be excluded completely.

In conclusion, we found that radiographic OA occurred more frequent in the previously injured knee joint than in the contralateral knee. The odds for developing radiographic knee OA were increased threefold after medial or bicondylar TPF compared to lateral unicondylar fractures and about fivefold in subjects who had OA in the contralateral knee. Patients with OA in the index knee had lower scores in both condition-specific and generic patient-reported measures than patients without OA, which indicates that TPF may contribute to the development of both OA disease and illness. Our results give sufficient background to identify patients at risk of developing posttraumatic OA. Those patients should be informed about the possible outcome and consequently employ an appropriate prevention strategy.

## Data Availability

Raw data for datasets are not openly available due to reasons of sensitivity and are available from the corresponding author upon reasonable request. Data are located in controlled access data storage at the Umeå University.
